# Microbial Therapy and Breast Cancer Management: Exploring Mechanisms, Clinical Efficacy, and Integration within the One Health Approach

**DOI:** 10.3390/ijms25021110

**Published:** 2024-01-16

**Authors:** Charalampos Filippou, Sophia C. Themistocleous, Giorgos Marangos, Yiannis Panayiotou, Maria Fyrilla, Christina A. Kousparou, Zoi-Dorothea Pana, Constantinos Tsioutis, Elizabeth O. Johnson, Andreas Yiallouris

**Affiliations:** School of Medicine, European University Cyprus, 6 Diogenis Str., 2404 Engomi, P.O. Box 22006, Nicosia 1516, Cyprus

**Keywords:** microbiome, breast cancer, microbial diversity, microbiota-based cancer therapies, one health, microbiome immunomodulation

## Abstract

This comprehensive review elucidates the profound relationship between the human microbiome and breast cancer management. Recent findings highlight the significance of microbial alterations in tissue, such as the gut and the breast, and their role in influencing the breast cancer risk, development, progression, and treatment outcomes. We delve into how the gut microbiome can modulate systemic inflammatory responses and estrogen levels, thereby impacting cancer initiation and therapeutic drug efficacy. Furthermore, we explore the unique microbial diversity within breast tissue, indicating potential imbalances brought about by cancer and highlighting specific microbes as promising therapeutic targets. Emphasizing a holistic One Health approach, this review underscores the importance of integrating insights from human, animal, and environmental health to gain a deeper understanding of the complex microbe–cancer interplay. As the field advances, the strategic manipulation of the microbiome and its metabolites presents innovative prospects for the enhancement of cancer diagnostics and therapeutics. However, rigorous clinical trials remain essential to confirm the potential of microbiota-based interventions in breast cancer management.

## 1. Introduction

The early detection of cancer is associated with fewer adverse effects and an increase in the success rate of treatments. Cancer diagnostic methods range in accordance to the level of invasiveness and many still require invasive biopsies for verification. Therefore, research is focused on developing diagnostic methods that are less invasive yet retain high sensitivity (identifying actual cancer cases) and specificity (excluding non-cancerous cases). The receiver operating characteristic (ROC) curve is a tool utilized in assessing the specificity and sensitivity of novel diagnostic tests. Measuring the area under the ROC generates a score, where a score of 1.0 symbolizes the ideal classification with 100% specificity and sensitivity [1].

Recent studies focus on employing samples such as saliva, stool, and plasma, where microbiome populations are documented as a non-invasive diagnostic approach, either directly or indirectly related to cancer. These procedures are less invasive than the current practices involving the collection of biopsies. Microorganisms play an indirect role in affecting the emergence, natural course, and/or severity of various cancers. For instance, microbes in the gut influence dormant cancer cells that may cause disease recurrence [2]. Predominantly, research connecting microbes and cancer has focused on bacteria and their involvement in different cancers, with a few studies also exploring potential links with fungi and viruses [2]. For example, research has indicated that metabolites from certain archaea, mainly from the *Euryarchaeota* phylum and the TACK superphylum, are linked to numerous cancers, despite these microbes being typically overlooked due to their rarity.

The presence of a unique microbiome in breast tissue, previously unacknowledged, has gained recognition through recent research. Microbiome dissimilarities have been observed between healthy and cancerous breast tissue, implying that cancer may disturb the natural balance of the microbiome in this area. Interestingly, breast tumor tissue showed a decrease in total bacterial DNA, and an inverse relationship was observed between the bacterial DNA load and advanced cancer stages [3]. Over 60% of breast cancer (BC) samples harbored bacterial expression both inside BC cells and immune cells. The microbial diversity in breast tumor samples surpassed that of other cancers and exhibited specific variations based on the receptor status. From these samples, live bacteria from phyla like Proteobacteria, Firmicutes, and Actinobacteria were isolated. *Fusobacterium nucleatum*, previously linked to colorectal cancer, was also found to have higher prevalence in breast tissue and colonized mammary tissue, enhancing tumor growth and metastasis.

An increase in *Methylobacterium radiotolerans* and a decrease in *Sphingomonas yanoikuyae* were also noted in tumor tissue [4]. Moreover, the fungal genus Malassezia was abundantly found in breast tumors [5]. Studies in mice have demonstrated the role of the local microbiome in the breast tissue microbiome in BC. Specific BC mouse models, introducing *Helicobacter hepaticus*, increased the presence of tumors in the mammary glands, mammary tissue inflammation, and the load of neutrophils expressed. An understanding of the typical microbiome of breast tissue and its transformation during BC might unveil microbes as potential therapeutic or preventive targets. A decline in bacteria within tumors was linked with fewer lung metastases. Staphylococcus spp. and Lactobacillus spp., in particular, were related to an upsurge in metastatic tumors. Furthermore, the expression of these microbes in the gut influences the onset and advancement of BC. However, further research is crucial to ascertain the universality of these microbial alterations across diverse patient groups [6,7].

In this review, we emphasize the ways in which the microbiome can both directly and indirectly influence various facets of breast cancer management, ranging from disease onset and progression to diagnostic and treatment options. We delve into recent studies that have reshaped our understanding of these interactions and their potential implications in cancer care.

## 2. Breast Microbiome in Breast Cancer Pathogenesis and Early Detection

The human microbiome is capable of influencing various human biological, hormonal, and metabolic pathways. This influence may in turn trigger the initiation, proliferation, and genetic instability of cancer within the host cell or may trigger apoptosis [8].

Evidence supports that gut microbiota dysbiosis affects the development, progression, and prognosis of BC both through direct and indirect signaling pathways. The microbial therapy of BC involves managing microbiota that will target the immune response. Studies found that the colonization of *Propionibacterium* and *Staphylococcus* was abundant in healthy control, high-risk, and tumor-adjacent normal tissue, but notably declined in tumor tissue [9]. The *Staphylococcus* genus, for example, is negatively associated with the expression of TRAF4, an oncogene that triggers a cascade of events favoring cancer survival through NF-κB [10]. The increasing expression of the *Staphylococcus* genus in BC tissue eliminates TRAF4 activity, alternatively increasing the activation of T-cell genes associated with the microbe. Equally, *Propionibacterium* expression is positively correlated with the activation of T-cells through NFAT2, NFIL3, and IFNGR, but its expression in BC tissue is reduced compared to healthy cells [10]. This study also presents the coexistence of *Methylibium* with multiple genes, including ICOS, MRC1, and Toll-like receptors, but this fact requires further investigation.

Normal breast tissue is characterized by the enrichment of six genera, including anaerobic *Lactobacillus*, which is associated with a progesterone receptor (PR)-positive status, and *Acetobacter*. Conversely, tumor tissue demonstrates a significant decrease in the abundance of these bacteria. Four primary bacterial species, specifically *Lactobacillus paracasei*, *Lactobacillus vini*, *Acetobacter aceti*, and *Xanthomonas* sp., are predominantly found in normal breast tissue. Meanwhile, a synergistic interaction between *Acetobacter* and *Lactobacillus*, resulting in the modulation of nutrient availability and a reduction in hist triglycerides, has been documented in the gut of *Drosophila melanogaster* [9,10,11,12]. The administration of *Lactobacillus acidophilus* increases cytokine expression such as IFN-γ in splenocytes [13]. This cytokine in turn increases the proliferation of lymphocytes such as NK cells, promoting type 1 T-helper activity, leading to anti-tumor immunity and anti-angiogenic effects [13]. TGF-β is an additional cytokine highly expressed by tumors and is involved in blocking T-cell production; this ensures that tumors are unaffected by immune surveillance. The expression of this cytokine is hindered by *L. acidophilus*; as a result, it reduces the tumor growth rate and increases lymphocyte proliferation.

Transferring *Micrococcus luteus* in in vivo models hinders 4TI tumor growth, and this may be due to the microbiota upregulating M1-macrophage-related genes, which include, among others, the IL-6, IL-8, and IL-12 genes [14]. The introduction of *M. luteus* peritumorally could improve patient outcomes; however, before reaching this stage, further safety and effectiveness evaluations are necessary. *Sphingomonas yanoikuyae* affects the immune response through the proliferation of invariant NKT cells involved in immunosurveillance, as well as the expression of TLR2, -5, and -9 [3]. Toll-like receptor 5 (TLR5) expression is also associated with microbial expression, e.g., *Salmonella typhimurium* flagellin, and activates the innate immune response in BC patients, marking TLR5 as a therapeutic target (Table 1) [15]. Moreover, the absence of inflammation in BC patients is caused by the absence of *Roseburia inulinivorans* in the microbiota, a case that is more common in postmenopausal BC patients than premenopausal BC patients [16].

*Escherichia coli* produce antimicrobial peptides that are activated under cellular stress conditions and affect cell survival through cell cycle arrest and DNAse activity [31,32]. Colicin A, for example, increases apoptosis in multiple breast cancer cell lines (MCF7, MDA-MD-231, MRC5, and osteosarcoma cell line HOS) by 7–28%, while Colicin E1 increases apoptosis even more in MCF7 (58%) and in HS913T cells (14%), whilst Colicin A is ineffective [32]. Anti-cancer expression also involves the membrane disintegration of BC cells following expression by a defensin peptide expressed by *Brevibacillus* sp. (Table 1) [33].

Another focus revolves around *Bifidobacterium*, whose anaerobic status suggests that it is ideal for the successful delivery of therapeutic genetic material to the necrotic centers of tumors. A plasmid has been assembled carrying a DNA-binding protein and *E. coli* as a source of cytosine deaminase required to convert the prodrug 5-Fluorocytosine to the tumor-toxic 5-Fluorouracil [34]. *Bifidobacterium* sp. also tackles cancer indirectly, through the metabolism of lapachol, synthesizing cytotoxic metabolites against BC, as proven in SKBR-3 cell lines [35]. This bacterium is also effective in synergy with *Bacteroides* (Table 1). The mixture of the two microbiota exhibits anti-breast-cancer properties as they suppress angiogenesis, proliferation, and apoptosis against BC cells [36]. The combination of these two microbiota also increases the secretion of interferon γ, inducing tumor cell lysis.

The histologic grading of breast tumors is associated with distinct microbial profiles. For example, invasive ductal carcinoma (IDC) and invasive lobular carcinoma (ILC) have unique microbial signatures. The status of estrogen receptor (ER) and human epidermal growth factor 2 (HER2) correlates with the abundance of a particular microbiome. *Streptococcus* and *Odoribacter*, found in non-tumor tissue, produce compounds that inhibit tumor invasion or promote anti-tumor activity. Additionally, lymphovascular invasion and a node-positive status are linked with the decreased presence of *Oblitimonas*. Lymphovascular invasion is positively related to *Lactobacillus* and negatively to *Alkanindiges*, while a node-positive status is positively associated with *Acinetobacter* and *Bacteroides* and negatively with *Achromobacter*. These findings indicate that the bacterial profiles associated with the prognostic features of breast tumors are both shared and distinct, suggesting that the interactions between the breast microbiome and tumor are multifaceted and likely influenced by various factors. Furthermore, microbiota-derived bile acids, which accumulate in breast tumors, are linked with reduced proliferation, suggesting a potential area for future research in relation to the breast microbiome [9,10,12,14].

Overall, modifying or eliminating certain communities of microbes can reverse the environmental conditions favoring cancer existence and triggering apoptosis. Complementary to traditional BC therapies, restoring breast microbiota to their local natural concentrations does not only ensure a functional immune response but can also prevent host microenvironment interference with the cancer drugs administered or the metabolites that they react with.

## 3. Microbiome Dynamics in Cancer Management

The dynamic nature of the gut microbiome, combined with tumor heterogeneity, can lead to varied observations across scientific studies. Numerous reports highlight alterations in both the alpha and beta diversity of the gut microbiome across various cancers, including BC.

A study by Aarnoutse et al. (2022) identified a significant reduction in species richness (*p* = 0.042) within the gut microbiomes of estrogen-receptor-positive BC patients undergoing treatment with (neo)adjuvant chemotherapy drugs [37]. A similar trend was observed in patients undergoing chemotherapy, as documented by Bilenduke et al. (2022), and concentrations returned to baseline post-treatment [38]. Conversely, Horigome et al. (2019) found no notable shifts in gut microbiome composition between chemotherapy-treated BC survivors and non-chemotherapy-treated survivors [39]. This disparity was attributed to the fact that the patients had completed their chemotherapy at least two years before the study’s commencement, suggesting that any chemotherapy-induced dysbiosis may have been reverted. Various other studies, such as the CANTO trial, have reported increased α diversity post-chemotherapy. Wu et al. (2022) made a similar observation for neoadjuvant chemotherapy patients [40].

Depleted microbial richness is linked to intensified depression symptoms, an increased fear of cancer recurrence, and the onset of diarrhea in BC patients undergoing chemotherapy [37,38,41]. Chemotherapy-associated cognitive impairment (CACI) is a widely documented side effect in BC patients, with deficits manifesting in memory retention, processing speed, and visuospatial ability [40,42,43,44]. An array of chemotherapeutic drugs has been implicated in inducing oxidative stress within the brain and promoting inflammation markers within the central nervous system.

Another possible mechanism underlying CACI involves the gut–brain interaction mediated by the peripheral immune system. The “Gut–Immune–Brain Axis” (GBA) theory proposes a communication triad between the intestinal microbiome, the immune system, and the brain. Chemotherapy-induced dysbiosis may disrupt the intestinal barrier, leading to immune cell infiltration and the release of pro-inflammatory cytokines into the bloodstream [40]. These cytokines can penetrate the blood–brain barrier (BBB), inducing neuroinflammation, resulting in cognitive disturbances [45]. Several studies support this theory by identifying a correlation between elevated pro-inflammatory cytokines and CACI in BC patients [43,46].

On a positive note, certain bacteria, known for producing butyrate, demonstrate potential anti-inflammatory and anti-cancer properties. The abundance of such beneficial bacteria, including *Coprococcus*, *Ruminococcus*, and *Faecalibacterium*, was noted in BC patients devoid of neurotoxicity post-chemotherapy, pointing to their possible role in curbing neuroinflammation [40].

### 3.1. Radiotherapy

Radiation therapy, or radiotherapy, primarily damages the DNA of cancer cells to induce cell death. Typically, it is administered locally but can also be combined with other treatments. Notably, radiation therapy causes gastrointestinal discomfort as a side effect, even if the gastrointestinal region is not the area under treatment. This could be due to microbiome dysbiosis as a side effect [47].

Few studies have delved into the impact of radiotherapy (RT) or chemoradiation (CRT) on cancer patients’ gut microbiomes. Specifically, for BC, clinical studies detailing RT’s influence on the gut microbiome are scant, even though it is a standard treatment for the majority of BC patients. Gynecological cancer patients undergoing RT exhibited variations in gut microbiota richness, with a decrease in beneficial gut commensal Firmicutes and an increase in opportunistic pathogens like *Fusobacterium* [48]. Several studies corroborate these findings, revealing that reduced alpha diversity and gut microbiome dysbiosis post-RT and CRT often accompany gastrointestinal side effects or fatigue in various cancers.

In cases of RT, it is noteworthy that higher alpha gut diversity has been associated with the increased tumor infiltration of activated CD4+ T-cells, subsequently leading to better recurrence-free and overall survival rates in cancer patients. While animal studies are foundational in understanding the intricate interactions of radiation, bacteria, and fungi, only one study, as of now, has investigated these interactions in a BC mouse model [49]. This study highlighted the importance of both bacteria and fungi in modulating the outcomes of RT.

Furthermore, studies have shown a connection between gut bacteria and the effectiveness of radiation therapy in treating various cancers. Eradicating Gram-positive bacteria with antibiotics improved the radiation’s anti-tumor effects in melanoma, lung, and cervical cancer mouse models. However, reintroducing the metabolite sodium butyrate, produced by these bacteria, eliminated this benefit [50]. The complete removal of gut bacteria reduced radiation’s effectiveness in some cancer models, while gut fungi depletion enhanced it [49]. The greater expression of fungal sensor Dectin-1 correlated with poorer BC survival rates. Mice’s responses to radiation varied based on their microbiota, with *Enterococcaceae* and *Lachnospiraceae* being prominent in treatment-responsive mice. Leukemia patients with these bacterial families experienced fewer gastrointestinal symptoms post-radiation. Metabolites from these bacteria were linked to radioprotection. Although research is limited, the role of the gut microbiota in the radiation response emphasizes the need for further study [51].

### 3.2. Chemotherapy

Chemotherapy primarily involves the use of medicinal drugs to chemically treat cancer by interfering with cell division (mitosis). The effectiveness of chemotherapy differs across various types of cancer. Its main objective is to harm or overwhelm cancer cells to induce programmed cell death (apoptosis), and some chemotherapy drugs can also stimulate immune reactions. Since chemotherapy is administered throughout the body, it may also impact normal cells that undergo rapid division. Due to its systemic administration, chemotherapy affects the gut microbiome, which may in turn also influence the effectiveness of chemotherapeutic treatments [52].

Administering antibiotics to neutralize the gut microbiome decreased the effectiveness of chemotherapy drugs like cisplatin and oxaliplatin in treating lymphoma and colon cancer in mice, attributed to reduced reactive oxygen species (ROS) production. This indicates the necessity of a functional commensal microbiome for the success of these platinum-based treatments. Antibiotics also lowered oxaliplatin’s effectiveness in a specific colon cancer mouse model. Subsequent experiments revealed an association between certain microbes and the drug’s effectiveness. For instance, the presence of *Paraprevotella clara* was linked to a lack of response for oxaliplatin, while *B. fragilis* correlated with a positive response. The consequence of administrating cyclophosphamide was the movement of particular gut microbes like *Lactobacillus johnsonii* and *Enterococcus hirae* to the spleen and lymph nodes, initiating an immune response, in melanoma and sarcoma mouse models. Further research showed that the existence of *E. hirae* and *Barnesiella intestinihominis* in the gut could enhance cyclophosphamide’s activity and rejuvenate its effectiveness post-antibiotic administration.

Additionally, the CANTO trial documented significant changes in the gut microbiome composition throughout chemotherapy [40]. Favorable commensals like *Dorea formicigenerans* and *Methanobrevibacter smithii* archaea are commonly found in healthy individuals but increased in abundance in BC patients post-chemotherapy. *Coprococcus* and members of the *Ruminococcaceae* and *Eubacteriaceae* families were positively linked with a good prognosis and the absence of axillary lymph node metastasis. Conversely, harmful bacterial species such as certain *Klebsiella* and *Bacteroides* species, along with members of the *Lachnospiraceae* and *Clostridiaceae* families, were associated with axillary lymph node invasion and advanced BC stages after chemotherapy. In particular, the presence of *B. uniformis* was abundant in non-metastatic advanced-stage BC, which was then reduced post-chemotherapy. The high abundance of *Bacteroides* also correlated with non-responsiveness to trastuzumab treatment in HER2-positive BC patients [53].

A metabolite from the gut microbiome, indole-3-acetic acid, has been observed to enhance the effectiveness of FOLFIRINOX, a combination chemotherapy used in treating metastatic pancreatic ductal adenocarcinoma. This enhancement is due to the accumulation of ROS and a reduction in the cancer cells’ autophagy. The gut microbiome does not solely influence the response to chemotherapy; bacteria in and around the tumor itself also play a significant role. There is growing interest in exploring how these local bacteria impact the effectiveness of chemotherapy and contribute to drug resistance in cancer patients.

Studies have shown that the commensal bacterium *E. coli* has the ability to modify the effectiveness of various chemotherapy drugs. It increased the toxicity of some drugs, while reducing the toxicity of others, like gemcitabine, doxorubicin, and mitoxantrone. Further experiments involving *E. coli* and gemcitabine in a mouse model indicated a decrease in the drug’s ability to combat tumors. Likewise, both *Mycoplasma hyorhinis* and *E. coli* have been found to metabolize gemcitabine into an inactive form, rendering cancer cells resistant. Similarly, colon cancer cells expressing adequate *F. nucleatum* exhibited greater drug resistance when exposed to certain chemotherapy drugs. This implies that microbes local to the tumor and its microenvironment can substantially impact chemotherapy’s effectiveness. On the other hand, manipulating the lung microbiota using aerosolized antibiotics and specifically probiotics showed improvements in the efficacy of a chemotherapeutic drug, dacarbazine. Such findings underscore the potential of further exploring microbial interactions in various body regions and within the tumor to enhance the outcomes of chemotherapy treatments.

### 3.3. Cancer Immunotherapy and Microbiome Immunomodulation

**Figure 1:**
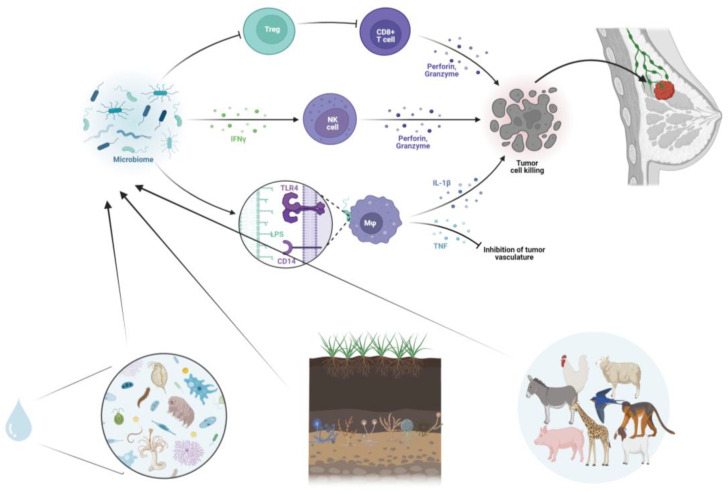
Microbiome and tumor microenvironment interplay. Schematic illustration of the microbiome-mediated modulation of the immune system, including Toll-like receptor activation, cytokine release, anti-tumor responses, inhibition of tumor vasculature, and immune cell activation.

Immunotherapy represents a prominent emerging therapeutic approach for certain hematological and solid malignancies, including BC (Figure 1). Bacterial metabolites directly influence the activities of local immune cells. These effects include the modulation of immunoglobulin secretion, the promotion of lymphocyte differentiation into regulatory T-lymphocytes and T-helper 17 cells, the generation of immunomodulatory cytokines, and even the epigenetic regulation of histone deacetylase enzymes.

Concerning the connection between the human microbiota and BC, various metabolites have been identified as potential risk factors or modifiers. These include substances such as estrogens, active phytoestrogens, short-chain fatty acids, lithocholic acid, and cadaverine. In particular, the gut microbiota’s production of estrogens, largely driven by the enzyme β-glucuronidase from specific intestinal bacteria, can result in the deconjugation of xenobiotics and sex hormones like estrogens. This process increases the reabsorption of estrogens into the systematic circulation, potentially elevating the risk of hormone-dependent BC in women. Conversely, some metabolites, such as phytoestrogens, lithocholic acid, and cadaverine, have been associated with a protective or risk-reducing influence on BC development [54].

Gut bacteria can also promote BC through the induction of chronic inflammation, which is closely linked to tumorigenesis. These bacteria, via pathogen-associated molecular patterns, can upregulate Toll-like receptors and activate NF-κB, a critical regulator of inflammation and cancer. NF-κB activation leads to the release of several cytokines, including IL-6, IL-12, IL-17, IL-18, and tumor necrosis factor-alpha, contributing to persistent inflammation within the tumor microenvironment [55]. Secondary metabolites released by intestinal bacteria, along with pro-inflammatory molecules reaching the liver via the portal vein, may further promote carcinogenesis. For instance, butyrate, a microbial metabolite, can enhance the anti-tumor cytotoxic CD8 T-cell response by modulating the ID2-dependent IL-12 signaling pathway [56].

The gut microbiome also plays a role in epigenetic deregulation, potentially affecting tumor development. Microorganisms produce bioactive substances with a low molecular weight, such as folates, short-chain fatty acids, and biotin, which can participate in epigenetic processes by altering substrates used for methylation or influencing the activity of epigenetic enzymes [57].

Immune checkpoint inhibitors are a class of therapies that leverage the immune system to combat tumors by blocking inhibitory interactions between T-lymphocyte receptors and ligands on malignant cells. While BC is typically not considered highly immunogenic compared to other malignancies like lung cancer or melanoma, recent data have shown the benefits of immunotherapy, particularly in the triple-negative subtype (ER/PR and HER2 negative).

Clinical trials have demonstrated varying response rates with different immunotherapies, alone or in combination with chemotherapy, in these patients. The KEYNOTE-012 trial investigated a pembrolizumab monotherapy in previously treated triple-negative breast patients, showing an overall response rate (ORR) of 18.5% and a median time to response of 17.9 weeks [58]. The KEYNOTE-086 trial tested pembrolizumab as a first-line therapy for metastatic triple-negative breast cancer, achieving an ORR of 23% [59]. Other trials, such as NCT01375842 and JAVELIN, assessed atezolizumab and avelumab, with observed ORRs of 10% and 5.2%, respectively [60,61].

Combinations of immunotherapy with chemotherapy have also been explored, with atezolizumab combined with nab-paclitaxel yielding an ORR of 67% in first-line treatment [62]. The IMpassion 130 trial examined the combination of atezolizumab and nab-paclitaxel in untreated metastatic triple-negative breast cancer patients, revealing a progression-free survival (PFS) benefit. The ENHANCE-1/KEYNOTE-150 trial evaluated eribulin combined with pembrolizumab, showing a higher ORR in PD-L1-positive BC patients [63]. The KEYNOTE-355 trial combined pembrolizumab with chemotherapy, demonstrating a significant progression-free survival benefit in patients with high PD-L1 values [64]. These trials collectively indicate the potential of immune checkpoint inhibitors in improving outcomes for triple-negative breast patients, particularly in cases with high PD-L1 expression (please see the summary box in Section 3.4).

### 3.4. Exploring Breast Microbiome Dynamics regarding Cancer Pathogenesis, Early Detection, and Management (Summary Box)

The human microbiome can influence pathways related to cancer initiation, progression, and genetic stability. Studies show that certain bacteria like Propionibacterium and Staphylococcus are abundant in healthy breast tissue but decline in tumors. Lactobacillus, Acetobacter, and others predominantly populate normal breast tissue. The administration of Lactobacillus acidophilus increases lymphocytes and cytokines, promoting anti-tumor immunity. The introduction of Micrococcus luteus in mouse models hinders tumor growth by activating M1 macrophages. Bifidobacterium may help to deliver therapeutic cargo to tumors. The breast microbiome composition differs between tumor subtypes and correlates with prognostic factors. Microbiome profiles linked to lymph node invasion or poorer prognosis involve Bacteroides or Lachnospiraceae. Escherichia coli peptides induce breast cancer cell apoptosis through cell cycle arrest and DNase activity. Colicin E1 increases apoptosis in MCF7 cells more than Colicin A. Brevibacillus sp. Expresses defensin peptides that disrupt breast cancer cell membranes. The gut microbiome’s dynamics influence cancer management. Studies show that chemotherapy reduces species richness and causes dysbiosis, linked to worse depression, cancer recurrence fears, and diarrhea. A possible mechanism for chemotherapy-associated cognitive impairment involves the gut–brain axis; chemotherapy-induced dysbiosis disrupts the intestinal barrier, allowing immune cell infiltration and pro-inflammatory cytokine release into the bloodstream and brain. Beneficial bacteria like Coprococcus may help to curb neuroinflammation. Radiotherapy impacts the gut microbiome, with decreases in Firmicutes associated with side effects. Gut bacteria and fungi modulation alter radiotherapy effectiveness in mouse models. Certain commensals enhance radiation responses. The microbiome influences chemotherapy effectiveness by metabolizing drugs or altering ROS production. Modulating the lung or tumor microbiota impacts chemotherapy responses. Immune checkpoint inhibitors show a benefit for triple-negative breast cancer, linked to microbiome immune modulation.

## 4. Microbiome-Modulating Interventions

### 4.1. Bacterial Therapeutics for Tumor Treatment and Immune Modulation

Microbial therapy is also termed oncolytic virotherapy or bacterial therapy. Alterations of certain microbial communities increase the risk of BC as they alter tissue metabolism on multiple levels, including the triggering and progression of cancer in the host [65]. This form of treatment may involve immune regulation, influencing the efficacy of anti-tumor drugs, the targeted therapy of engineered pro- and prebiotic fecal microbiota transplantation, and the administration of anti-tumor drugs [66].

The recent scientific discourse has highlighted the significance of the microbiota in mitigating BC via its anti-tumor activities [67]. Contemporary studies assert that specific intestinal bacteria can obstruct oncogenesis and promote tumor regression. Nonetheless, the indiscriminate modulation of the gut microbiota can manifest unforeseen consequences. The precise targeting of tumor-associated bacteria is thus crucial in ensuring the safety and effectiveness of therapeutic modalities [68,69]. Current research underscores the imperative of regulating specific tumor-inducing bacteria through methodologies such as bacteria-mediated tumor therapy (BMTT), fecal bacterial and bacteriophage transplantation, prebiotic enhancement, and the utilization of bacterial toxins and enzymes [70].

However, a salient concern in harnessing bacterial agents for cancer therapeutics pertains to their potential cytotoxicity and pathogenic manifestations. To mitigate these risks, the scientific community has explored genetic engineering as a solution, allowing the excision of virulence-inducing genes while preserving therapeutic features [71]. However, the selection of bacteria is paramount: an ideal bacterial candidate should effectively permeate tumors, possess minimal infectiousness, and remain amenable to antibiotic-mediated elimination post-intervention. Numerous bacterial candidates await rigorous clinical evaluations, leaving certain aspects of their metabolic functions ambiguous. Challenges encompass inadvertent cytotoxic effects on healthy cells, incomplete tumor eradication, and unpredictable bacterial genome mutations. The transient existence of bacterial peptides in the human body further complicates their therapeutic potential. As such, there is an emergent demand for exhaustive clinical trials to elucidate these bacteria–tumor cell dynamics and the potential ramifications of such interventions [72].

Conventional cancer treatments, primarily radiotherapy and chemotherapy, remain central to BC intervention paradigms. Despite their dominance, the associated adverse effects and non-selective toxicity of these treatments necessitate the exploration of more targeted therapeutic options. In this context, genetically engineered bacteria, which exhibit a selective affinity for cancer cells while sparing healthy tissue, have emerged as promising agents in cancer research [73].

These bacteria inherently possess the ability to produce and excrete proteinaceous toxins that can inhibit specific cellular functions, a property that may be harnessed for therapeutic purposes. These agents can target cancer cells, thereby minimizing damage to surrounding healthy cells. Studies have suggested a potential symbiotic relationship between certain bacterial strains and tumor regression, observed in animal models. Nevertheless, there is an unequivocal need for additional research to validate and understand these associations further [74].

While the exclusive use of bacterial agents may not secure complete tumor elimination, their combination with conventional drugs presents a promising avenue for cancer treatment. This integrated approach—incorporating drug-laden bacteria—appears especially propitious in overcoming the limitations of existing treatments, primarily their inability to effectively penetrate the tumor microenvironment [75,76].

The utilization of bacteria, particularly *Listeria*, *Clostridium*, and *Salmonella*, offers a solution to this challenge as they can navigate and infiltrate the tumor’s environment. For instance, *Salmonella typhimurium* has demonstrated remarkable anti-tumor properties, showing efficiency in invading and eliminating various cancer cells in in vivo studies. This strain has yielded significant results as a monotherapy against pancreatic, prostate, and BC in animal models [77,78].

Furthermore, *Clostridium perfringens* has been acknowledged for producing an enterotoxin that, upon interaction with specific transmembrane proteins, can induce tumor regression. The FDA, recognizing the potential of BMTT, approved the use of Bacillus Calmette–Guerin, a weakened strain of *Mycobacterium bovis*, for a specific bladder cancer treatment in the late 1970s, a testament to the enduring clinical relevance of this approach [79,80].

Despite these promising advances, bacterial-mediated therapies are not without challenges. Concerns like potential antibiotic resistance and infection risks associated with the use of live bacteria in treatments need careful consideration and resolution. Hence, while the paradigm of BMTT is not new in oncology, its widespread application and implementation remain the subjects of ongoing debate and investigation. The preliminary findings, although promising, underscore the importance of continued and expanded research to fully comprehend and validate the potential symbiosis between bacterial strains and tumor regression.

### 4.2. Probiotics and Prebiotics

Emerging data suggest a nexus between microbial dysbiosis in the breast and gut regions and the onset and progression of BC [81]. BC pathogenesis is frequently linked to sustained inflammation, incited by intestinal bacteria that activate NF-κB, subsequently releasing pro-inflammatory cytokines such as TNF-alpha [82,83,84]. Variations in the gut microbiome have been observed across different BC stages [85,86]. These alterations can influence the therapeutic efficacy and potential toxicity. Interventions aimed at modifying the gut microbiome, employing probiotics and prebiotics, could be instrumental in attenuating systemic inflammation and alleviating treatment-associated toxicity. Specific strains such as *Bifidobacterium* and *Lactobacillus* have shown promise due to their immunomodulatory and antigenotoxic characteristics [87]. Recent research by Yazdi et al. (2012) revealed that the prophylactic administration of *Lactobacillus plantarum* augmented with selenium nanoparticles led to a significant reduction in tumor volume and elevated survival rates in a murine model of advanced human BC [88]. Furthermore, milk fermented with *Lactobacillus casei* CRL 431 administered in the same model demonstrated a marked decrease in tumor invasiveness and metastatic potential [89].

In addition, there have been a multitude of clinical trials in the oncology field investigating the efficacy of probiotics alongside standard anti-cancer regimens (Table 1). These studies predominantly indicate a beneficial reduction in the gastrointestinal side effects that often arise from conventional cancer therapies [90,91,92,93,94,95]. A significant finding was obtained in RCC patients who received a bacterial supplement, CBM588, alongside immunotherapy, reporting improved progression-free survival rates and response outcomes [96]. Probiotics play a crucial role in enhancing the immune system, significantly elevating the immunoglobulin A (IgA) levels in the gut, which is vital for immune function. Specific probiotic bacteria, including *L. casei* and *Sphingomonas yanoikuyae*, have been noted to bolster the production of natural killer (NK) cells, playing a pivotal role in regulating cancer progression by actively participating in the body’s defense against cancer. Moreover, probiotics not only foster immune defenses but also produce compounds instrumental in protecting against DNA damage and breaking down carcinogens, thereby potentially preventing cancer. For example, *L. casei* strain Shirota (BLS) has been studied for its probable cancer-preventive properties, showing an inverse correlation between its consumption and the incidence of BC [97]. Furthermore, probiotics have shown promise in reducing chemotherapy-related cognitive impairment (CRCI) in BC patients and alleviating various other chemotherapy-induced side effects [98,99].

Species like *L. casei* Shirota and *Bifidobacterium* Bb12 have exhibited antigenotoxic activity, a crucial component in preventing the genetic mutations that may lead to cancer, although the degree of this effect varies between bacterial species and is dependent on long-term exposure. Furthermore, the effectiveness of immune cell activation by probiotics varies and is based on the dosage and bacterial strain.

However, despite the optimistic preliminary findings, it is essential to acknowledge that the effects of probiotics tend to diminish over time. This diminishing effect underscores the need for ongoing research to understand the long-term role and potential benefits of probiotics in both cancer prevention and treatment, and in mitigating the side effects associated with cancer therapies. Given the initial promising results, predominantly based on in vivo experiments, there is a clear need for enhanced, comprehensive research, including in vitro exploration, to overcome the challenges observed in existing studies, such as inefficient mucosal adhesion and reduced gastrointestinal activity.

Current clinical trials are sparse, especially those focusing on probiotics specific to BC. Nevertheless, the initial findings are promising, and the results from ongoing clinical trials are anticipated to provide a clearer understanding of the role that probiotics play in potentially enhancing BC treatment outcomes and in cancer prevention more broadly.

### 4.3. Bacteriotherapy Approaches

Bacteriotherapy, an evolving field in the domain of anti-cancer therapies, employs a diverse range of bacterial forms, inclusive of genetically modified organisms (GMOs), in both their living and attenuated states. These bacterial entities function via promoting apoptosis or disrupting cell membranes, primarily targeting cancer cells through a range of anti-cancer agents, including bacteriocins, spores, and bacterial peptides [100].

Historical records indicate the rudimentary utilization of bacteriotherapy as far back as 1550 BC. A seminal advancement in cancer immunotherapy can be attributed to Dr. William Coley, an American orthopedic surgeon. His pioneering work involved the development of a heat-inactivated concoction of *Streptococcus pyogenes* and *Serratia marcescens*, subsequently referred to as “Coley’s toxin”. Administering this therapeutic concoction to patients with inoperable cutaneous carcinoma yielded substantial clinical outcomes, notably tumor regression and complete remission in a significant proportion of patients [101].

Various bacterial strains, encompassing *Vibrio*, *Shigella*, *Salmonella*, *Listeria*, and *Bifidobacteria*, have shown profound efficacy in tumor invasion, colonization, and subsequent eradication. However, the relationship between bacteria and cancer remains multifaceted. Certain bacterial strains possess the potential to instigate carcinogenesis or induce malignancies. A case in point is *Helicobacter pylori*, which, through the secretion of specific cytokines and chemokines, can incite chronic inflammatory responses with detrimental cellular implications. Notably, the cytotoxin-associated gene A (*cagA*) has emerged as a pivotal bacterial protein with profound implications in oncogenesis, primarily by compromising the function of the tumor suppressor protein p53 [102,103].

Although, historically, malignancies were often associated with pathogenic bacterial infections, contemporary research is increasingly highlighting the anticarcinogenic potential of certain bacteria. This paradigm shift can be epitomized by the nuanced understanding of how tumor cells proliferate by subverting host immunologic defenses. Recent studies highlight the anticarcinogenic activity of *Salmonella*, which appears to operate through a multifaceted mechanism involving both adaptive and innate immune response activation.

Bacteriocins, intricate peptides or proteins synthesized ribosomally, first garnered attention in 1920, courtesy of the seminal work by the Belgian scientist André Gratia and the discovery of colicin (the first bacteriocin) from *E. coli* [104]. Today, their applications transcend the clinical domain, with their antimicrobial properties finding utility in food preservation as well. Based on their molecular weight, they are categorized into four classes. Significant bacteriocins include Bovicin HC5 from *S. bovis* and Nisin A from *Lactococcus lactis*. Some research indicates that the enterotoxin (TcdA) and cytotoxin (TcdB) produced by *Clostridium difficile* could be pivotal in treating colorectal cancer [105].

Bacteria, in their myriad forms, are increasingly being recognized as potent immunotherapeutic agents. Their ability to modulate tumor antigenicity holds promise in augmenting immune responses. The novel insights into bacterial interactions, particularly infections with *Clostridium novyi*, underscore the profound potential in harnessing bacteria for therapeutic advancements in oncological properties. Therefore, bacteria are hailed as promising immunotherapeutic agents due to their ability to amplify the antigenicity of tumor cells, thus bolstering immune responses. By using bacterial cancer immunotherapy, tumor cells are identified as infected cells rather than mere cancer cells, elevating the likelihood of their elimination. For instance, infections with *C. novyi* can trigger the formation of heat shock proteins like Hsp70, promoting the maturation of professional dendritic and antigen-presenting cells, leading to potent antigen-specific immune responses [106,107].

### 4.4. Fungal Microbial Polysaccharides

Microbial polysaccharides (MPs) play a role in various cellular processes, including signal transduction and immune response modulation [108]. Glycans and Basidiomycetes-derived MPs, like krestin, schizophyllan, and lentinan, exhibit anti-cancer properties through immunostimulation, the downregulation of NF-κB responses, and the induction of tumor cell apoptosis [109,110].

Polysaccharide peptides from the mushroom YunZhi (*Coriolus versicolor*/*Trametes versicolor*) have been used in combination with chemotherapy to treat BC patients in Asian countries for decades (Table 1). These peptides exhibit anti-proliferative properties, as they significantly reduce BC cell (MDA-MB-231) proliferation by upregulating the p21 gene expression [111]. Moreover, a meta-analysis by L.Y. Eliza et al. (2012) concluded that *Coriolus versicolor* can increase survival rates in cancer patients, including those with BC [112].

Additional beneficial MPs include Levan, which induces apoptotic cell death in MCF-7 BC cells, and the proteoglucan D-Fraction from *Grifola frondosa*, which reduces mammary tumor cell migration and lung metastases [113,114]. A clinical trial on D-Fraction in breast and lung cancer patients (stage II–IV) found that it hindered cancer progression and metastasis and increased NK cell activity [115]. This suggests MPs’ potential in enhancing anti-tumor immunity in BC patients without significant toxicity. However, there is a need for additional clinical trials to study D-Fraction’s efficacy in combination with chemotherapeutics in a larger patient population.

### 4.5. Oncolytic Virotherapy

Oncolytic virotherapy (OV) employs either naturally occurring or genetically engineered viruses with an affinity for tumor cells, leading to their selective targeting and replication. This process culminates in tumor regression, attributed not only to direct cytotoxicity but also the stimulation of anti-tumor immune responses. Remarkably, this occurs without negative effects on healthy cells and tissue [116].

In both in vitro and in vivo preclinical investigations, the third-generation oncolytic herpes simplex virus-1 (HSV-1) vector, G47Δ, exhibited amplified cytotoxic effects across multiple BC cell lines, inclusive of MCF-7, MDA-MB-468, and the tamoxifen-resistant variant MCF-7/TAM-R [117,118,119]. Notably, a synergistic cytotoxic effect was observed in BC cells when G47Δ was combined with the chemotherapeutic agent paclitaxel. This potentiated the anti-tumor efficacy of paclitaxel, resulting in a five-fold dosage reduction to achieve an equivalent tumor reduction in vivo [119], a shift that could minimize chemotherapy-associated adverse effects.

In 2015, the U.S. Food and Drug Administration (FDA) granted approval for talimogene laherparepvec (T-VEC) as the inaugural oncolytic agent for melanoma treatment. Derived from the genetically modified HSV-1, its utility was further highlighted in a phase II clinical trial, which revealed that, in conjunction with neoadjuvant chemotherapy (NAC), it enhanced pathological complete response rates in triple-negative breast cancer (TNBC) patients, resulting in an 89% 2-year disease-free rate [120]. It warrants mention that the observed adverse effects, though generally mild, ranged from injection site pain and headaches to low-grade fevers. This was notably in contrast to the more severe immune-mediated toxicity associated with TNBC treatment involving both chemotherapy and pembrolizumab [121].

GLV-1h68, an oncolytic vaccinia virus, has been engineered to selectively target and destroy cancer cells. Studies found that this virus replicated more efficiently in ALDEFLUOR-positive BC cells, which are known for their resistance to chemotherapy and radiation and their higher expression of cancer stem cell markers. These cells also demonstrated greater migration and invasion abilities. In mouse models, tumors derived from these cells responded more robustly to GLV-1h68 treatment, showing earlier fluorescence detection and faster regression. The virus also showed preferential replication in another cancer stem-like population, CD44+CD24+ESA+ cells. This efficient infection and destruction of BC stem-like cells by GLV-1h68 highlight its potential as a promising agent against tumor initiation, recurrence, and metastasis [122].

Similarly, the measle virus secretory form of NAP (MV-s-NAP), an oncolytic measles virus, has been engineered to express secretory neutrophil-activating protein (s-NAP) from *H. pylori*, which is an immunostimulatory bacterial protein (Table 1). This virus selectively targets and destroys cancer cells, particularly cancer stem-like cells. It also triggers both local and systemic anti-tumor immunity. s-NAP attracts immune cells like neutrophils and macrophages to the site of infection and induces the expression of pro-inflammatory cytokines and chemokines. This activates both the innate and adaptive immune responses against the infected tumor cells. In preclinical studies, MV-s-NAP demonstrated improved efficacy over the MV alone, doubling the survival time in mouse models of BC and increasing the levels of certain cytokines like TNFα, IL-6, and IL-12/23 in the pleural effusion. The virus was well tolerated in transgenic mice, with no adverse effects observed, supporting its favorable preclinical safety profile. These promising results have led to the initiation of a first-in-human phase I clinical trial for metastatic BC (Table 1) [21].

### 4.6. Phage-Based Immunotherapy

Likewise, phage therapy is emerging as a promising strategy in BC immunotherapies. It utilizes the ability of phages to invoke anti-tumor immune responses. Specifically, in phage display immunotherapy, antigens (proteins or peptides), fused to phage coat proteins, function as protective vaccines against cancer. Certain peptides, including E75, AE37, and GP2, have shown potential in BC tests on BALB/c mice. Phages offer two main vaccine delivery techniques: (i) showcasing immunogenic peptides through modified phage coat proteins and (ii) acting as delivery media for DNA vaccines by inserting a eukaryotic promoter-driven vaccine gene within their genome. These vaccines, presenting numerous antigen copies on immunogenic phage particles, incite strong immune reactions. They are also stable, cost-effective, and potent. Experiments have validated their effectiveness in mice and rabbits. Vaccines against human papilloma viruses (HPV), such as Gardasil-9, demonstrate applications beyond BC. Another anti-breast cancer development is a phage-based anti-HER2 vaccine, designed to bypass immune tolerance. Other studies have developed vaccines for prostate cancer and utilized inovirus-associated vector vaccines for antibody production. Furthermore, a dual anthrax-plague vaccine and a lambda phage-based vaccine for hepatocellular carcinoma underscore the expansive potential of phage-based therapies.

Additionally, in a recent study by Catala and colleagues (2021), protein-lipid particles (PLPs) have been innovatively crafted using bacteriophage lambda to display a fluorescent probe and the therapeutic antibody trastuzumab (Trz), leading to the formation of Trz-PLPs [123]. These are designed to target HER2-positive BC cells. By increasing Trz’s density on PLPs, the more prolonged inhibition of cell growth is achieved compared to using free Trz. Trz-PLPs have impacts on numerous cellular pathways, influencing amino acid metabolism, mitochondrial function, and more. They modulate the phosphorylation of key signaling proteins, such as Akt and mTOR, influencing the vital PI3K/Akt/mTOR signaling pathway in cancer cells [123]. Dong and colleagues (2022) have also spotlighted the potential of the M13 phage-based vaccines [124]. By combining an M13 phage with a cationic polymer, PEI, a hybrid platform (M13@PEI) was designed, capable of efficiently absorbing negatively charged antigens. This resulted in the MPO vaccine, combined with the Ovalbumin (OVA) antigen, which improved the maturation of antigen-presenting cells (APCs) and boosted antigen presentation. The M13 phage genome’s CpG regions and PEI’s role as a TLR5 agonist facilitated this. Enhanced antigen processing and uptake were further confirmed through in vitro assays, emphasizing the robust cytotoxic T-lymphocyte (CTL) response. In vivo studies also confirmed the MPO vaccine’s ability to deliver antigens effectively and enhance antigen-specific T-cell-mediated responses. When combined with α-PD1 treatment, the vaccine showcased powerful anti-tumor effects, improved survival rates, and enhanced immune memory responses, proving the significant potential of M13 phage-based vaccines in anti-tumor immunotherapy [124] (please see the summary box in Section 4.7).

### 4.7. Microbiome-Modulating Interventions (Summary Box)

Bacterial therapeutics show promise for tumor treatment and immune modulation. Specific gut bacteria can prevent cancer development, while others promote it, so precisely targeting tumor-associated bacteria is important. Approaches include bacteria-mediated tumor therapy, fecal transplants, bacteriophages, and using bacterial toxins/enzymes. However, risks include cytotoxicity and pathogenicity, which can be mitigated through genetic engineering. Ideal bacterial candidates effectively reach tumors with low infectivity and can be eliminated with antibiotics. Probiotics and prebiotics also show potential by reducing inflammation and treatment side effects. *Lactobacillus plantarum* and *Lactobacillus casei* have demonstrated anti-tumor effects in mouse models. Clinical trials show that probiotics can reduce chemotherapy side effects. Specific strains like *L. casei* and *Sphingomonas* increase natural killer cells and immune defenses against cancer. However, the effects tend to diminish over time, underscoring the need for long-term research. Bacteriotherapy employs living or attenuated bacteria to promote apoptosis, disrupt cell membranes, or deliver drugs specifically to cancer cells. Approaches include genetically modified organisms, bacteriocins, spores, and peptides. Historical examples include Coley’s toxin, which induced remissions. *Salmonella*, *Listeria*, and *Clostridium* can penetrate tumors. However, concerns include antibiotic resistance, infection risks, and uncertain metabolic functions. Fungal polysaccharides also exhibit anti-cancer properties through immunostimulation, inflammation reduction, and apoptosis induction. Mushroom extracts have shown activity against breast cancer in Asian studies and mouse models. Oncolytic virotherapy employs viruses, like HSV-1, to selectively target and replicate in tumors, inducing direct killing and immune responses against cancer cells. Several viruses are under clinical investigation for breast and other cancers. Phage display vaccines also show potential by presenting tumor antigens on phage coat proteins to stimulate immune responses.

## 5. Integrating One Health Approach in Cancer Ecology

**Figure 2:**
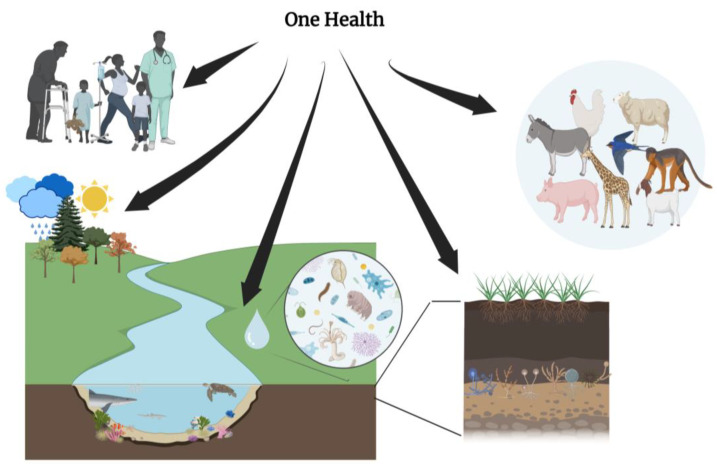
One health conceptual diagram. One Health includes human health and well-being, animal health, and environment health, as well as the microbial and biochemical ecology of soils and plants. The interconnectivity and dynamic interaction between all of them emphasizes that the level of each can affect the other, stressing the need for their balance.

The holistic approach to health promoted by the One Health (Figure 2) principles mainly focuses on infectious diseases and zoonoses [125]. However, the mechanisms underlying oncogenesis and novel management strategies in oncology recognize the interrelations between human health, animal health, and the environment, thus placing an emphasis on the transdisciplinary and multisectoral relevance of One Health. The connection between environmental, animal, and human health and oncogenesis is supported by several observations [126,127]. One is the transmissibility of specific malignancies between animals: although rare, the transmission of cancer between individuals of the same or related species (such as the canine transmissible venereal sarcoma) has been documented [128]. In addition, the transmission mode in several cases remains undefined (e.g., in the case of a leukemia-like disease among marine bivalves, such as clams and mussels [128]). Similarly, from an evolutionary perspective, the transmission of cancer between different species remains a possibility [128,129]. Evolutionary cancer suppression mechanisms have been detected in animal species: one such example is the development of immune-modulating resistance against devil facial tumor disease (DFTD) reported in Tasmanian devils (*Sarcophilus harrisii*), which probably contributed to the survival of the species over this fatal type of transmissible cancer [130]. Another example is that of the myxoma virus (MYXV) and the European rabbit (*Oryctolagus cuniculus*), which co-evolved, selecting for viruses of attenuated virulence that caused extended (over fatal) disease and, in parallel, for rabbits that developed resistance to myxomatosis [131] through genetic alterations that facilitated an enhanced immune response to the disease [132]. Such recorded cases provide evidence that transmissible cancers can exert selection pressure on the affected species, promoting the domination of organisms with genetic alterations and immune mechanisms that confer a survival benefit. The mutagenetic and oncogenetic ability of human activities to animals is also relevant: the extent, intensity, and nature of human activity have significant impacts on the diversity of the environment and on the health and habits of different animal species, resulting in variable contact patterns between animals, arthropods, and humans and facilitating the emergence and re-emergence of infectious diseases [133]. Simultaneously, it is well established that environmental alterations resulting from urbanization and differences in air, soil, noise, light, water pollution, environmental degradation, and dietary interventions precipitate oncogenic processes in humans, thus increasing the risk of cancer. The accumulating literature suggests that these conditions also affect the health of animals, including wildlife, through various pathways, such as endocrine and immune dysregulation, chronic inflammation, nutritional disruptions, oncogenic infections, epigenetic changes, chromosomal alterations, and microbiome changes [134,135,136].

The shared environment between animals and humans affects the microbiota composition and microbiome, unraveling possible causative relationships with cancer development. An organism’s homeostasis and physiology are closely intercalated with its microbiome. Similarly, environmental perturbations, anthropogenic or not, can influence the microbiota of both humans and animals, with health implications across different organs and systems [137,138,139].

In animal studies, westernized diet-associated bacteria exerted transgenerational effects in utero, resulting in chronic disease and cancer in mice [140]. Furthermore, antibiotic-affected microbial communities in bees led to significant alterations in gene expression in the host, which could also be passed down through generations [141]. Such findings signify the close relationship between the microbiome, normal host functions, genetic changes, and the ability to pass these changes across generations.

Taken together, these observations advocate for collaborative efforts to address the various mechanisms underlying cancer development by acknowledging the similarities between the animals and humans sharing an ecosystem. Acknowledging the disruptions and imbalances based on the host microbiota composition, through the physiologic and homeostatic changes instigated by disease and the environment, a One Health approach to cancer consists of the following.

Surveillance: cancer surveillance across human medicine, veterinary medicine, and environmental science. Areas and species can serve as sentinel targets for the early detection of carcinogens in the environment and for cancer patterns that might reflect eco-environmental exposures or cancer types that warrant further research.

Detection, including early warning systems: interdisciplinary collaboration to create diagnostic tools (e.g., biomarkers, screening protocols, geographic information systems). The employment of artificial intelligence and machine learning tools can help to combine surveillance, diagnosis, and warning systems to facilitate early diagnosis and prompt detection.

Elucidating and addressing common risk factors: several risk factors for cancer, such as exposure to carcinogens and lifestyle choices (e.g., nutrition, physical activity), affect both humans and animals [142]. Understanding the presence and pathophysiologic mechanisms of shared risk factors can help to elucidate oncogenetic pathways or contribute to the design and implementation of preventive strategies. Surveillance and prompt detection can facilitate the design of future studies in order to elucidate unidentified risks for cancer development in different species [143].

Environmental health: the association between the environment and disease is bidirectional. The behavior of animal species can increase the cancer risk through the induction of cancer-associated environmental exposures. Spatial, temporal, ecological, and population changes can have documented and unpredicted effects on the emergence, re-emergence, and frequency of diseases [133,142]. These are not limited to communicable diseases, but can span across endocrine conditions, malignancies, cardiovascular diseases, metabolic conditions, skin conditions, toxin exposures, etc. [144]. Up to one fourth of global deaths and one fifth of disability-adjusted life years (DALYs) in humans are associated with environmental exposures [144]. Conversely, environment-attributable diseases can affect the behavior of animals, leading to additional environmental changes. For example, poor living conditions in rural areas drive urbanization, further increasing air and noise pollution in urban areas. In wildlife, diseased animals are more likely to fall to prey, thus increasing the transmission risk of communicable diseases, while representing an inferior nutritional source for preying [37] species. Animals and humans who are malnourished or suffer from chronic disease have a higher risk of other diseases after exposure, either to environmental factors, pollutants, or transmissible agents, increasing the incidence of environment-associated diseases and affecting the growth, behavior, and health of animal populations [142]. Cumulatively, environmental degradation increases the risk of disease, and diseased populations can affect the environment, creating a vicious cycle of eco-environmental health [145].

Cancer prevention: prevention strategies span over different levels, including primordial, primary, secondary, tertiary, and quaternary [145]. Health promotion, a principle focusing on humans, focuses on empowering people to increase control over their own health [146]. In the context of One Health, cancer prevention extends beyond the individual and populational levels of health to the broad geospatial and interspecies framework of ecosystems [142].

Antimicrobial stewardship: an important and emerging aspect of One Health, the ecological effects of antimicrobials at the population and environmental level are globally recognized. Exposure to antimicrobials facilitates the dominance of resistant bacterial populations in the environment, acquiring genes that have the potential to be transmitted in the environment, from organism to organism and between species [147]. Antimicrobial usage has been found to be directly related to the number of resistance genes in the gut microbiomes of humans, even in the absence of direct antimicrobial administration [148]. Water and soil constitute significant environmental reservoirs of antimicrobial-resistant genes [149]. Antimicrobial-resistant genes can spread in the environment to such an extent that they can even reach air masses in areas of high antimicrobial usage [150]. These observations highlight the urgent need for stringent efforts to control the transmission of antimicrobial resistance in the community. The strongest risk for the emergence of antimicrobial resistance in community settings is the inappropriate use of antimicrobials in humans, animals, and agriculture [149]. Indeed, the extent of the inappropriate use of antimicrobials in animal husbandry, veterinary medicine, and agriculture significantly exceeds their use in human medicine, representing a key target for stewardship interventions [151]. Antibiotic-associated tissue dysbiosis has been associated with tumor development, further supporting the need for the prudent use of antimicrobials in animals [152]. In addition, the disruption of normal immune responses to malignant cells, induced by antimicrobials, can have detrimental outcomes in organisms with cancer, by affecting their responses both to cancer and to therapeutic agents [153].

Ethical considerations: factors related to ethical issues surrounding animal welfare, humane treatment, healthy livelihoods, access to optimal healthcare, and experimental studies, for all living organisms, are integrated into the One Health approach, ensuring that research, medical interventions, and environmental health are addressed under the same moral boundaries [146,154,155] (please see the summary box in Section 5.1).

### 5.1. Integrating One Health Approach in Cancer Ecology (Summary Box)

The One Health approach recognizes the connections between human, animal, and environmental health. Cancer development can be influenced by factors that impact all three domains. Some cancers can even be transmitted between animals or between species. Changes to the environment from human activities can impact wildlife health and increase cancer risks by altering genetics, immune function, and microbiomes. Shared risk factors like toxic exposures also exist. Surveillance across human and veterinary medicine can help to detect new carcinogens and cancer patterns related to the environment. Understanding common risks can help to design prevention strategies. Prudent antimicrobial use is important to curb resistance, as antibiotics can disrupt microbiomes and immunity in ways that influence cancer. Ethical considerations regarding animal welfare must also be integrated. Overall, the One Health perspective supports a collaborative, multi-sectoral approach to uncovering cancer causes and advancing prevention, detection, and treatment strategies.

## 6. Conclusions and Future Directions

Employing agents involved in multiple human biological reactions, such as the immune response and metabolism, may be key to developing tumor drugs with higher selectivity and specificity, hence reducing adverse side effects for patients. These drugs could be prescribed in synergy with traditional cancer treatments, which include radiation, immunotherapy, or chemotherapy. These forms of treatment can potentially diminish drug resistance. A microbiome screen test can pinpoint potential targets that may help to improve patient prognosis, as previously described, but may also avert drug resistance. Additionally, standardized protocols need to be composed to ensure that clinical guidelines and protocols are available. Such a uniform system will also allow for the collection of efficiency and safety evidence. Breast tumors can be accessed either by adjusting the gut microbiota or by introducing microbiota transport vectors peritumorally. Ultimately, the relationship between the gut and breast microbiome colonies requires additional evaluation prior to designing new drugs that work in this way and an evaluation of the effects of the vector microbiomes on the immune system.

### 6.1. Integration of Microbial Therapy within the One Health Approach

The concept of One Health describes a transdisciplinary approach considering the interaction between plants, animals, people, and the environment that they share. The basis of this concept is to recognize and eliminate zoonotic diseases, water contaminants, antimicrobial-resistant germs, vector-borne diseases, and more. Evidence so far supports that there is a strong link between the microbiome and cancer development, cancer proliferation, and apoptosis. Specific human microbiomes also serve as biomarkers for certain cancers. Shifting the microbiome (expression) accordingly reverses these effects and it does so through a more holistic approach [156]. Manipulating the gut microbiota through such an approach makes for more personalized treatments with fewer side effects and potentially greater health outcomes. This could also improve patients’ mental positions as they may appreciate the concept being offered as a more holistic treatment option compared to chemical drugs.

Interdisciplinary collaboration between human and animal researchers can advance microbial research by providing epidemiological insights into the disease. A partnership between veterinary medicine and human medicine can increase the amount of data available on cancer and the behavior of microbiota and provide valuable information that can enable the progress of therapeutic methods. As comparative and spontaneous oncology emerge, more information becomes available as to how an animal’s immune system fights off neoplasms. The extrapolation of this information can increase our understanding of the behavior of a cancer and hence provide novel therapeutic targets and treatment schemes [157]. More importantly, as the ownership of both domestic and exotic pets is increasing, the need to survey One Health elements is crucial to monitor and potentially prevent outbreaks.

Transmissible cancers are emerging, accelerating the loss of biodiversity across ecosystems and redirecting research as to why these cancers arise and how they unfold and prevent any potential pandemics [127]. An additional aspect that requires further evaluation is the degree to which malignancies in animals, such as BC, are transmissible to humans.

Identifying the exact microbiome species through genetic analysis can uncover their environmental origin, whether animal or other food products. Chemical agents also disturb the microbiome. Fertilizers provide crops with nonspecific nutrients, meaning that microbiota populations are also nourished and hence proliferate; once produce is consumed by humans, the gut microbiota is subsequently increased [158]. Infant feeding, infections, medication, and diet are among the environmental factors that need to be further assessed to further understand the impact that they could have on BC. An ongoing study (NCT03885648) is evaluating the hypothesis that one risk of BC for humans could be environmental contaminants affecting the mammary/gut microbiota.

### 6.2. Regulatory Considerations and Ethical Implications

Despite emerging microbiome-based therapies showing promising therapeutic results in BC therapy, both regulatory and ethical considerations must be thoughtfully evaluated. This is to ensure patient safety and diagnosis. Prior to marketing a drug for microbiome-based therapy, clinical trials are essential to evaluate the drug’s safety and efficacy, followed by the evaluation of the results from the FDA and European Medicines Agency (EMA). After the marketing of drugs, off-label use by clinicians may also be a factor to consider in terms of both regulatory and ethical concerns, ensuring that the patient is constantly informed of the efficacy and limitations of the treatment that they are receiving. It is critical to ensure, with informed consent, that patients are fully aware of the limited knowledge available on the long-term effects of administering microbiome therapies [159]. Microbiomes can possess both tumor suppressor and promoter properties; therefore, interventions causing dysbiosis must be investigated to identify how genes and lifestyle (e.g., diet) affect the natural biodiversity of the microbiome. This information will also affect patient responses to microbial therapy. Access to microbiome therapies is also of ethical concern, as it is important to ensure that individuals from different populations and socioeconomic groups all have access to these drugs. The utilization of big data and machine learning for precision medicine is already being discussed. This raises data privacy concerns as the use of omics technologies will involve databases that store and constantly analyze sensitive data on the gut microbiomes of multiple individuals. The setup such data sets calls for adherence to ethical guidelines and approval from ethical committees and requires the completion of informed consent forms [160].

### 6.3. Future Directions

The suitable regulation of the gastrointestinal microbiome by antibiotics or dietary regimes restrains estrogen’s effects and regulates bacterial activities, which in turn can prevent BC or BC reoccurrence. As microbes translocate to the mammary tissue from the gut, they also affect the inflammatory response [161]. Further gene analysis is required to map all commensal microorganisms and other wild-type microorganisms naturally expressed in the human body that contribute to the additional genetic material necessary for human metabolism. The focal aspects that need to be determined prior to a microbiome being labelled as a therapeutic agent require us to first identify which microbiota species are naturally present in the human body under ‘healthy’ conditions and their concentrations, and finally the levels at which these microorganisms stimulate harmful responses.

Mapping out all microbes that inhabit a patient’s body can provide insights for the cancer treatment plan. As previously mentioned, the microbiota in both the gut and within the tumor microenvironment can affect drug metabolism and disturb the activity of chemotherapeutic agents [162]. By reversing dysbiosis, the microbiome can be reverted to the known ‘healthy’ levels, avoiding tumor drug resistance and subsequently the harmful effects instigated by these drugs. Dietary plans may also accompany drug administration for more efficient results; for instance, in vivo results have shown that hyperbaric oxygen therapy accompanied by a ketogenic diet has significant anti-tumor effects [163].

Proceeding with microbial therapies will also require supplementary investigation to understand how microbiome-to-microbiome interaction can affect patient prognosis. This is necessary as the same drug can behave differently in every human individual due to the unique composition of their microbiome [164]. Additional factors exerting a shift in microbiome concentrations should also be considered, as they may affect a patient’s diagnosis and prognosis. These factors include age, the stage of the cancer, and whether the patient is in a pre- or postmenopausal state [165]. Evidently, *Lachnospiraceae* and *Clostridium* are abundant in stage II and III BC patients’ gut microbiomes as compared to stage 0 and stage I. Postmenopausal BC patients show a greater presence of pathogenic bacteria than premenopausal patients, proposing a non-invasive approach, as these concentrations can be rereferred to as markers for detection and prevention, and potentially therapeutic targets [166].

*Fucobacterium nucleatum* is an anaerobe that suppresses T-cell accumulation while promoting tumor growth and increasing metastasis [167]. Additional evaluation as to whether eliminating the microbiota will have the opposite effect and allow for anti-cancer cells to react effectively is needed. An additional pathway requiring further exploration is the use of *E. coli* secretum as biomarkers for BC, as they disrupt key metabolic pathways of MCF-7 cells [113]. As microbial settlements are volatile, continuous monitoring is essential to ensure that suitable adjustments are made to increase the treatment efficacy. Clinical trials are necessary to further evaluate the effectiveness and safety of these therapies both short and long-term. The latter is especially crucial provided that some microbiota are key for normal biological functions; thus, altering their concentrations or introducing them in other parts of the human body than their normal environment could have unintentional consequences.

### 6.4. Promising Avenues for Further Research and Development

Bacteriotherapy has been a possibility since 1988 and involves the implantation of gut flora from a healthy individual to a patient, reversing gut dysbiosis to improve the diagnosis [168]. Bacteria have been enlisted in the treatment regimes of multiple cancers: *Salmonella typhimurium* for colon and breast carcinoma, *E. coli* for nasopharyngeal carcinoma, *Klebsiella pneumoniae* for cervical cancer, *Pseudomonas aeruginosa* in multiple cancers, and *C. botulinum* in BC [169,170]. Due to their anaerobic properties, microbiota can enter a tumor microenvironment without being affected by its necrotic and hypoxic properties; hence, one mode of action involves utilizing the microbiome as a vector to deliver chemotherapeutic drugs deep into the tumor itself, so that they may exert their apoptotic tumor effects [171]. An alternative mechanism involves microbiome expression tuned to ‘healthy’ levels, permitting chemotherapeutic drugs to perform their anti-cancer effects successfully within the human body without interference and with limited adverse side effects. Having determined the fluctuations that the microbial community concentrations undergo due to the presence of a tumor, novel biomarkers can be targeted for tumor diagnosis. The future diagnosis of cancer may involve the completion of genomic profile screening on a patient biopsy to identify the dysregulated microbes that need to be targeted. These findings can then be utilized to establish an effective patient-specific cancer-microbial therapy plan with the least side effects.

Microbial therapy where fecal microbiota transplantation is involved will require the detailed screening of the donor to ensure that the material to be retrieved is free from alternate diseases or even infectious agents that could affect the patient. Provided that the materials are extracted from another human donor, this may also lead to unexpected immunological side effects within the patient. Finally, similarly to other novel treatments, the long-term safety is unknown.

In this review, we explore the influence of both the gut and local microbiomes on various aspects of cancer care, encompassing development, progression, diagnosis, and treatment. Recent advancements have seen groups harnessing synthetic biology to modify commensal microbes, triggering specific immune responses against diseases, including cancer. As research techniques and analytical models evolve, our understanding of the microbiomes within and around us will deepen. We foresee a marked rise in the discovery of microbes associated with cancer, moving beyond the readily culturable. These newly discovered microbes represent promising avenues for the refinement of diagnostic and therapeutic strategies, setting the stage for enhanced cancer prevention and more promising treatment outcomes. Additionally, interdisciplinary collaborations between human, animal, and environmental health researchers will provide invaluable insights into the cancer–microbiome interplay. Comprehending the identity, distribution, and activity of human microbes in both health and disease, through a One Health approach, is essential in strategically modulating the microbiome and harnessing its intricate ties with BC for improved patient outcomes.

## Figures and Tables

**Table 1:** List of the clinical trials posted and their current status. The databases searched included ClinicalTrials.gov (US) and the EU Clinical Trials Register; the studies with the following status were excluded: “Unknown Status”, Terminated”, “Closed without recruitment”, or “Withdrawn”.

Potential Therapeutic Microbe	Clinical Status	Clinical Phase	Type of Cancer	Mechanisms Involved	References
*Bacillus*	Recruiting	N/A	Breast cancer stage I–III	NCT05717972: Evaluate the effects of a fermented tea, kombucha. Once brewed, sugar breaks down from the SCOBY and probiotic bacteria such as *Bacillus coagulans*, *Bacillus subtilis*, and *Lactobacillus rhamnosus*, among others, are released.	[17]
*Bacteroides*	Active, not recruiting	Phase II	Weight loss in breast cancer patients	NCT04499950: Compare how the ratio between Bacteroides and Firmicutes in microbiome affects weight loss in breast cancer patients.	[18]
*Coriolus versicolor/Trametes versicolor*	Completed	Phase I	Lung neoplasms and breast carcinoma	NCT02603016: Patients will be given maitake mushroom (*Grifola frondosa*) extract to evaluate treatment efficacy.	[19]
	Completed	Phase I	Breast cancer	NCT00680667: To determine the side effects and effective dose at which the muschroom extract *Coriolus versicolor* is effective in treating women with breast cancer between stages I and III following radiation therapy. Results not posted.	[20]
*Helicobacter pylori*	Recruiting	Phase I	Recruiting	NCT04521764: Genetically engineered measles virus expressing *Helicobacter pylori* neutrophil-activating protein is administered to breast cancer patients to determine shredding and immune response rate and to record any side effects and therapeutic doses.	[21]
*Lactobacillus*	Not yet recruiting	Phase 2 or phase 3	Invasive ductal carcinoma (IDC) or invasive lobular carcinoma (ILC)	NCT04362826: Evaluate efficacy of the BIOHM probiotic synthesized of *B. breve*, *S. boulardii*, *L. acidophilus*, and *L. rhamnosus* microbes. Determine whether any bacteriome and mycobiome profiles of breast tissue are altered following consumption of BIOHM and determine whether quality of life is different for patients that have consumed the probiotic.	[22]
	Completed	Early phases of clinical trial	Breast adenocarcinoma	NCT03358511: Patients diagnosed with a breast adenocarcinoma administered a dietary supplement known as Primal Defense Ultra^®^ (bearing *Saccharomyces*, *Lactobacillus* and *Bifidobacterium*). Postmenopausal breast cancer patients took the probiotic 3 times a day for 2–4 weeks prior to surgery. Results not yet posted.	[23]
	Not yet recruiting	Early phases of clinical trial	Breast cancer patients stage I–III	NCT06039644: Stage I-III breast cancer patients undergoing anthracycline-based and taxane-based chemotherapy are instructed to consume probiotics (various *Lactobacillus* strains) over a 6-month period to determine whether chemotherapy side effects are improved or even prevented.	[24]
	Completed	Phase 2 or phase 3	Breast cancer patients with vaginal flora score on Nugent scale IV–VI	NCT01723592: Probiotic capsules enclosing four lyophilized *Lactobacillus* strains were prescribed as a dietary supplement to patients. The study was designed to determine whether the vaginal flora could be improved by at least 2 grades on the Nugent scale. Results indicated that probiotics effectively reduced the Nugent score and were more effective when prescribed during chemotherapy.	[25]
	Completed	Early phases of clinical trial	Greater than 25% risk of developing breast cancer (but have never had breast cancer) and/or BRCA1 or BRCA2 positive)	NCT03290651: The hypothesis is that *Lactobacilli* can restore the breast microbiome of women, displacing any harmful cancer-causing bacteria, and reduce inflammation.	[26]
*MV-s-NAP*	Recruiting	Phase I	HER2 breast cancer	NCT04521764: Study investigating the side effects and therapeutic dose of MV-s-NAP (modified measles virus) for breast cancer. Laboratory work has shown that the virus abolishes breast cancer cells.	[21]
*Salmonella*	Completed	Phase I	Patients with advanced or metastatic cancer	NCT00004988: *Salmonella typhimurium* (VNP20009) administered to nonresponsive metastatic melanoma or renal cell carcinoma patients. Observed tumor colonization with no anti-tumor effects.	[27]
Not stated	Recruiting	Phase IV	Breast cancer patients with vulvovaginal atrophy	NCT05562518: Through patient-reported outcome measurements, the efficacy of the following combinations of drugs will be evaluated and compared to determine which treatment plan provides the best quality of life for vulvovaginal atrophy in breast cancer. Drug: Estrogen Drug: Dehydroepiandrosterone Drug: Estrogen + probiotics Drug: Moisturizer	[28]
According to patient stool analysis	Ongoing	Phase III	Triple-negative breast cancer	EudraCT Number: 2017-002771-25 (NCT03281954). Investigate the gut microbiome population prior to cancer treatment and 30 days following the last treatment to identify the role of gut microbiota in regulating immune response. The impact of the gut microbiota on cancer incidence and its progression will also be evaluated.	[29]
Probiotic regimen to be designed according to patient stool analysis prior to treatment	Completed	Early phase I	Breast cancer stage I–III and breast adenocarcinoma	NCT04857697: Breast cancer patients will be administered probiotics, prior to surgery, to determine whether it affects patient outcomes. Dysbiosis and immune system effects will also be evaluated.	[30]

N/A: This study is not in clinical phase.

## Data Availability

Not applicable.
